# Presence of *O*‐glycosidically linked oligosaccharides in the cell wall mannan of *Candida krusei* purified with Benanomicin A

**DOI:** 10.1002/2211-5463.12558

**Published:** 2018-12-10

**Authors:** Takuya Kuraoka, Akito Ishiyama, Hiroko Oyamada, Yukiko Ogawa, Hidemitsu Kobayashi

**Affiliations:** ^1^ Laboratory of Microbiology Department of Pharmacy Faculty of Pharmaceutical Science Nagasaki International University Sasebo Japan; ^2^ Sasebo City General Hospital Japan

**Keywords:** antibiotic, Benanomicin A, *Candida krusei*, cell wall mannan, *O*‐linked sugar, β‐elimination

## Abstract

Cell wall mannan of the pathogenic yeast *Candida krusei* was prepared using the antibiotic Benanomicin A, which has a lectin‐like function. The chemical structure of this molecule was found to be similar to that of mannan prepared from the same yeast by the conventional method using Fehling reagent. Only a few degradation products were detected when the mannan prepared using Fehling reagent was subjected to alkali treatment (β‐elimination), but multiple α‐1,2‐linked oligosaccharides were detected when the mannan purified with Benanomicin A was treated with alkali. These results indicate that most of the *O*‐linked sugar chains in mannan were lost under conventional conditions when exposed to the strongly alkaline Fehling reagent. In contrast, the *O*‐glycosidic bond in mannan was not cleaved and the *O*‐linked sugar chains were maintained and almost intact following treatment with the mild novel preparation method using Benanomicin A. Therefore, we argue that the new mannan preparation method using Benanomicin A is superior to conventional methods. In addition, our study suggests that some yeast mannans, whose overall structure has already been reported, may contain more *O*‐linked sugar chains than previously recognized.

**Abbreviations**
Fr. K‐B: *C. krusei* mannan purified by Benanomicin method
Fr. K‐F: *C. krusei* mannan purified by Fehling method
NMR: nuclear magnetic resonance
M: d‐mannose residue
M2: biose
M3: triose
M4: tetraose

Invasive candidiasis represents the most common invasive fungal infection in the developed world. Mortality among patients with invasive candidiasis is very high 1. *Candida albicans* is the main pathogen of invasive candidiasis; however, during recent years, the rate of invasive candidiasis due to non‐*albicans Candida* species has increased 1, 2, 3. Two *Candida* species, *C. krusei* and *C. glabrata*, produce resistant strains against azole antifungal agents 4, 5. Accurate diagnosis is necessary to treat these infections with appropriate antibiotics, but rapid and easy diagnostic methods for identifying the species of genus *Candida* have not yet been clinically developed. One method for the identification of *Candida* yeast species is to detect a species‐specific gene. In addition, since the detection methods of genes are extremely sensitive, one risk is that *Candida* yeast species present in healthy people will also be detected. Therefore, another rapid diagnostic method for mycoses including endogenous infection may involve a classical immunochemical technique using an antigen–antibody reaction.

Tsuchiya *et al*. 6, 7, 8 previously analyzed the cell wall polysaccharide antigen of fungi including yeast of genus *Candida*. They revealed the presence of some specific antigens to fungi species and showed that these were useful for the identification of fungi species. Mannan is most important in the interaction between yeast and its host since this molecule is present in the outermost layer of the cell wall. For this reason, structural analysis studies of cell wall mannan of pathogenic *Candida* species have been an active area of research, and the overall structure and antigenic determinants of this molecule have been reported in several clinically important *Candida* species 9, 10.

One important requirement for the antigen analysis of *Candida* yeasts is the isolation of intact mannan molecules responsible for its antigenic activity. The most common procedure for purifying yeast‐derived mannan is by precipitating this molecule after it forms a complex with copper in Fehling reagent. We previously isolated various mannans from several pathogenic *Candida* yeasts by this procedure and analyzed the *N*‐linked polysaccharide moiety of these molecules and partial structure corresponding to various antigenic determinants 11, 12, 13, 14, 15, 16, 17. However, the use of the conventional mannan purification method using a strongly alkaline Fehling reagent results in a loss of *O*‐linked sugar chains from mannan. In fact, very small amounts of oligosaccharides can only be obtained using a dilute alkali treatment (β‐elimination) of mannans from various *Candida* yeast purified by the Fehling reagent 18, 19. As a result, most structural and immunochemical studies on yeast mannans so far have only mentioned the *N*‐linked sugar moiety of the molecule.

In the 1980s, a screen was performed by a pharmaceutical company that focused on polysaccharides specifically present on the surface of fungal cells, such as mannan, β‐glucan, and chitin. As a result, Benanomicin A, which has antifungal and anti‐HIV activities, has been isolated from the culture solution of *Actinomadura sporax* 20, 21, 22. The chemical structure of Benanomicin A (molecular weight, 864), an extremely dark red compound, is a glycoside composed of benzo [a] naphthacenequinone, d‐alanine, and a disaccharide (Fig. 1). Benanomicin A initiates antifungal action by selectively binding to the mannose residue of yeast cell wall mannan in the presence of Ca^2+^ 23. Therefore, Benanomicin A is regarded as an equivalent to lectin in its function even though it is not a protein.

**Figure 1:**
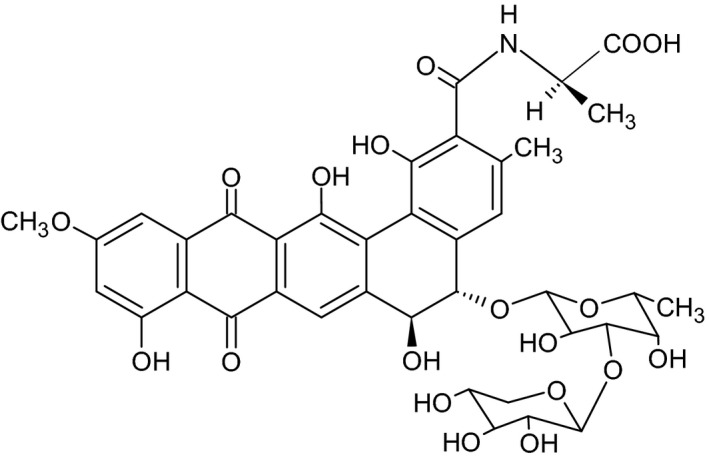
Chemical structure of Benanomicin A.

As mentioned above, *C. krusei* is a clinically important pathogenic yeast, but its immunochemical properties and chemical structure of its cell wall mannan remain unknown 7. The purpose of this study was to prepare cell wall mannan of *C. krusei* under rapid and mild conditions using Benanomicin A. We then demonstrated the presence of alkaline labile sugar chains in this molecule. These experimental results were then compared with those of conventional refining methods using the Fehling reagent.

## Materials and methods

### General

*Candida krusei* NBRC 0584 strain was obtained from the National Institute of Technology and Evaluation, Chiba, Japan. The yeast‐form cell of this strain was cultivated in Sabouraud liquid medium [0.5% (w/v) yeast extract, 1% (w/v) peptone, and 2% (w/v) glucose] for 48 h on a reciprocal shaker.

Benanomicin A was kindly provided by Dr. Shuichi Gomi (Pharmaceutical Research Center, Meiji Seika Kaisha, Ltd., Tokyo, Japan).

### Preparation of the crude extract of *Candida krusei* NBRC 0584 strain

The cells were harvested by centrifugation and washed with aqueous 0.9% (w/v) NaCl and then dehydrated with large volumes of acetone. The aqueous suspension of acetone‐dried cells was subjected to heat extraction at 130 °C for 3 hours. After cooling, the brown gel was concentrated *in vacuo* to a thick syrup and was dialyzed against running tap water for 48 h. The retentate was then concentrated *in vacuo* to a thin syrup, and the volume was adjusted to 20 mL with water. This solution was then lyophilized. The yield of the mannan crude extract was 18.5% (w/w) based on the acetone‐dried cell weight.

### Purification of *Candida krusei* mannan by using Benanomicin A

**Figure 2:**
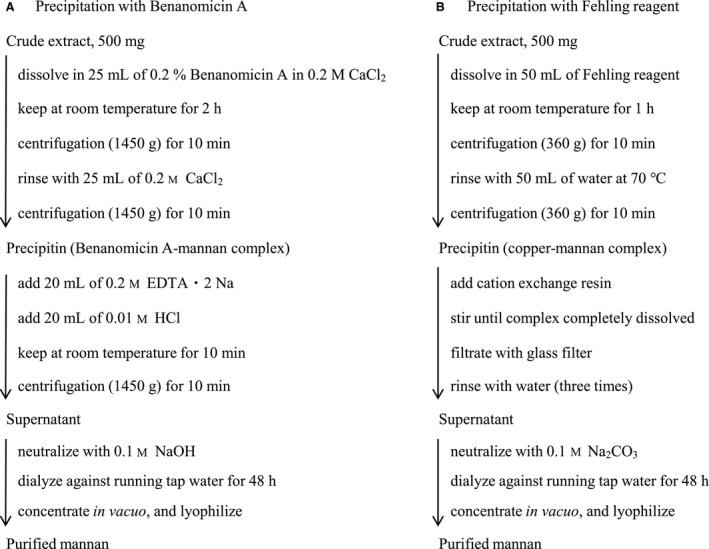
Procedure of Benanomicin method (A) and Fehling method (B).

Preparation of mannan using Benanomicin A was carried out following Fig. 2A. First, 25 mL of 0.2% (w/v) Benanomicin A in 0.2 m CaCl_2_ was added to the solution in which 500 mg of crude extract was dissolved, and under vigorous stirring. After 2 h, the resultant red precipitate was collected by centrifugation at 1450 ***g*** for 10 min, and the residue was rinsed with 25 mL of 0.2 m CaCl_2_ under vigorous stirring. The precipitate (Benanomicin A–mannan complex) was transferred to a 100‐mL beaker. Next, 20 mL of the aqueous solution of 0.2 m EDTA•2Na and mixed with 20 mL of 0.01 m HCl was mixed with the precipitate. After 10 min, the precipitin (Benanomicin A and EDTA‐Ca•2Na chelate) was removed by centrifugation at 1450 ***g*** for 10 min. The supernatant was neutralized with 0.1 m NaOH, dialyzed against running tap water for 48 h, concentrated *in vacuo* to 5 mL, and then lyophilized. The above‐mentioned method is referred to as the Benanomicin method, and *C. krusei* mannan purified by this method is abbreviated as ‘Fr. K‐B’.

### Purification of *Candida krusei* mannan by the conventional method using Fehling reagent

As shown in Fig. 2B, mannan was prepared using Fehling reagent as previously described 24. Fehling reagent consists of a 1 : 1 (v/v) mixture of 3.5% CuSO_4_ 5H_2_O, 17.3% Rochelle salt and 5.0% NaOH. This method is referred to as the Fehling method. *Candida krusei* mannan purified by this method is abbreviated as ‘Fr. K‐F’.

### Treatment of Frs. K‐B and K‐F with alkali (β‐elimination)

Frs. K‐B and K‐F were dissolved in 30 mL of 0.1 m NaOH, respectively, and the solution was incubated at 25 °C for 18 h. Then, the solution was neutralized with 1 m HCl, concentrated to a small volume, applied to a Bio‐Gel P‐2 column (2.5 × 100 cm) (Bio‐Rad, Tokyo, Japan), and eluted with water (0.25 mL·min^−1^).

### Other methods

Total carbohydrate content was determined by the phenol‐H_2_SO_4_ method 25 using d‐mannose as a standard. Total protein content was determined by the Folin method 26 using bovine serum albumin as a standard. Total phosphate content was determined by the method of Ames‐Dubin 27 using KH_2_PO_4_ as the standard. The ^1^H‐NMR spectra were recorded by means of a JEOL JNM‐AL400 spectrophotometer in D_2_O solution at 70 °C, using acetone as the standard (2.217 ppm).

## Results

### Establishment a purification method of mannan by using Benanomicin A

In order to establish a purification method for mannan from the yeast cell wall utilizing the lectin‐like activity of antibiotic Benanomicin A, various conditions were examined (data not shown). Consequently, we propose a new method as shown in Fig. 2A. This method does not require exposure to a strong alkaline environment like the conventional Fehling method (Fig. 2B). Thus, the Benanomicin method not only provides mild conditions for the purification of mannans, but also reduced the procedure time (Fig. 2A).

### Chemical analysis of Frs. K‐B and K‐F

Yields of Frs. K‐B and K‐F were 54.7 and 30.1% (w/w), respectively, based on the crude extract. This fact indicates that *C. krusei* mannan molecules bind efficiently to Benanomicin A rather than Fehling regent, resulting in the formation of a precipitate. The chemical compositions of Frs. K‐B and K‐F are shown in Table 1. Phosphorus was not detected in any fraction, but both K‐B and K‐F fractions contained small amounts of protein. The protein content of Fr. K‐B was higher than that of Fr. K‐F, suggesting that the mild conditions of the Benanomicin method resulted in less damage to the protein.

**Table 1:** Chemical compositions of *Candida krusei* mannan fractions. K‐B and K‐F

Mannan	Total carbohydrate^a^ (%)	Total protein^b^ (%)	Total phosphate^c^ (%)	Yield^d^ (%)
Fr. K‐B	88.8	9.1	0	54.7
Fr. K‐F	94.9	3.4	0	30.1

a Determined by phenol‐H_2_SO_4_ method using d‐mannose as a standard.

b Determined by BCA protein assay using bovine serum albumin as a standard.

c Determined by Ames‐Dubin method using KH_2_KO_4_ as a standard.

d Weight based of crude extract.

### ^1^H‐NMR analysis of Frs. K‐B and K‐F

**Figure 3:**
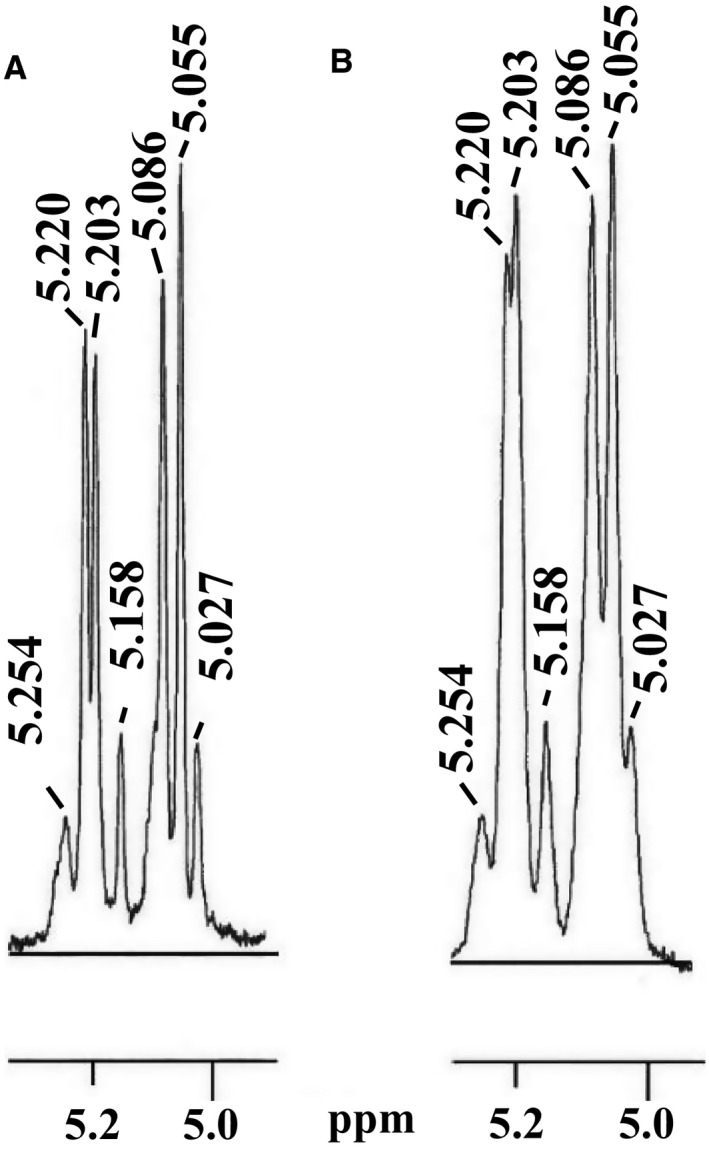
^1^H‐NMR spectra (anomeric region) of *Candida krusei* mannan, Frs. K‐B (A) and K‐F (B).

To obtain information on the sugar chain structure, which is a major component of Frs. K‐B and K‐F, ^1^H‐NMR analysis was carried out (Fig. 3). The spectra of both fractions showed extremely similar patterns in the anomeric proton region (range of 4.7–5.7 ppm). This finding suggests that the new purification method of yeast mannan was just as effective as conventional methods. The absence of any signal in the range of 5.40–5.70 ppm indicated that both fractions did not contain mannose residues via a phosphodiester bond. Four strong signals derived from α‐1,2‐ and α‐1,6‐linked mannose residues (5.220, 5.203, 5.086, and 5.055 ppm), and three weak signals derived from an α‐1,3‐linked mannose residue (5.254, 5.158, and 5.027 ppm) were observed in both fractions. In summary, based on our previous report 12, it became clear that cell wall mannan of *C. krusei* cell does not contain a β‐1,2‐linked mannose residue and phosphodiester bond.

### Alkali treatment (β‐elimination) of Frs. K‐B and K‐F

**Figure 4:**
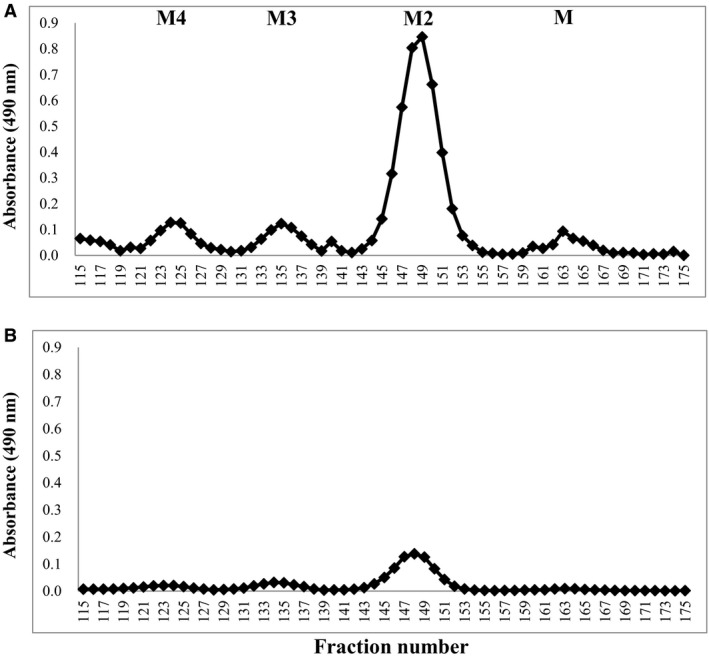
Elution profiles of oligosaccharides obtained from Frs. K‐B (A) and K‐F (B) by the alkali treatment (β‐elimination). M4, M3, M2, and M indicate the eluted positions of standard mannooligosaccharides, tetraose, triose, biose, and mannose, respectively.

**Figure 5:**
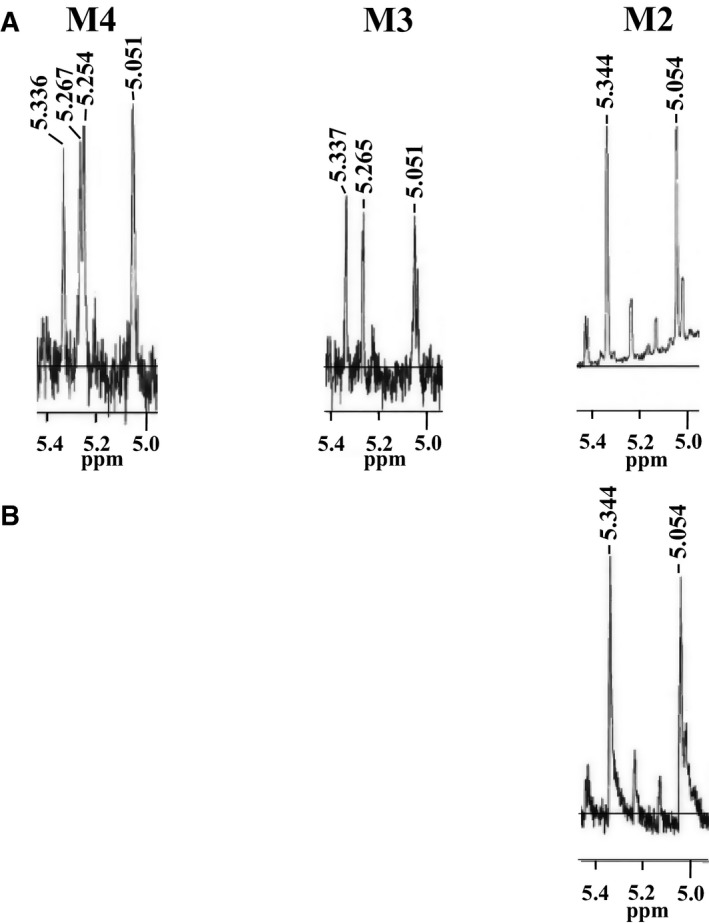
^1^H‐NMR spectra of oligosaccharides obtained from Frs. K‐B (A) and K‐F (B) by the alkali treatment (β‐elimination). M4, M3, and M2 indicate tetraose, triose, and biose, respectively.

As shown in Fig. 4, to identify alkaline labile sugar chains in *C. krusei* mannan, Frs. K‐B and K‐F were subjected to dilute alkali treatment, and their products were fractionated by gel chromatography. The yields of oligosaccharides released from Frs. K‐B and K‐F were 20.0 and 1.4% (w/w), respectively. This finding proves that most of the *O*‐glycosidic sugar chains in the *C. krusei* mannan molecule were lost at the purification stage by the Fehling method. In other words, the Benanomicin method can be used to isolate mannan in their original form without hydrolysis of *O*‐glycosidic bonds. The products of Fr. K‐B after the alkaline treatment consisted of low‐molecular‐weight oligosaccharides, tetraose (M4), triose (M3), and biose (M2), as well as minimal amounts of mannose. On the other hand, in the same treatment of Fr. K‐F, only biose was able to collect an analyzable amount in the next NMR. The ^1^H‐NMR spectra of these oligosaccharides are shown in Fig. 5. Based on our previous findings 28, we determined that all of these oligosaccharides were composed only of α‐1,2‐linked mannose residues. Namely, M4, M3, and M2 were identified as Manα1‐2Manα1‐2Manα1‐2Man, Manα1‐2Manα1‐2Man, and Manα1‐2Man, respectively.

## Discussion

**Figure 6:**
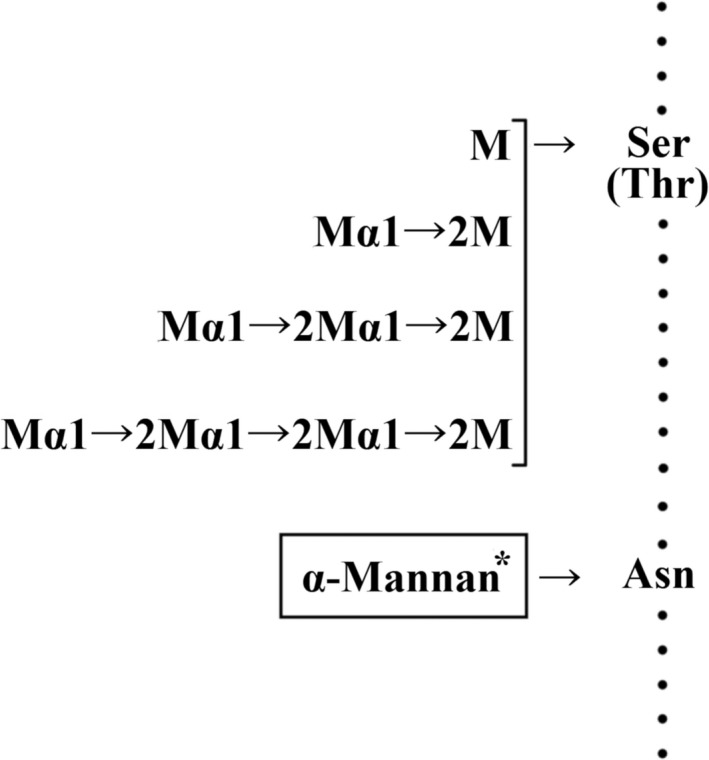
Structure of *O*‐linked sugar chains in the cell wall mannan of *Candida krusei*. Ser, Thr, Asn and M indicate serine, threonine, asparagine and mannose, respectively. *The chemical structure of *N*‐linked sugar chains in this mannan is not clear yet.

In this study, we determined that sugar chains linked to the hydroxyl group of serine and/or threonine in the mannan–protein complex (mannan) derived from *C. krusei* NBRC 0584 were low‐molecular‐weight oligosaccharides composed only of α‐1,2‐linked mannose residues (Fig. 6). In addition, the chemical structure of the sugar chains linked to the amino group of asparagine of this mannan was composed of α‐1,2‐, α‐1,3‐, and α‐1,6‐linked mannose residues. Although the carbohydrate domain of *C. krusei* mannan appeared to be composed of about 20% *O*‐linked sugar chains and about 80% *N*‐linked sugar chains (Table 1), the detailed chemical structure of this *N*‐linked sugar chain has not been reported yet. Our unpublished research data showed that the *C. krusei* mannan structure was distinct from the comb‐like structure presented by mannans of other *Candida* species and therefore further studies are required.

Previous immunochemical studies of pathogenic *Candida* yeast mostly targeted *N*‐linked sugar chains of cell surface mannan. In these previous reports 9, 10, 29, the Fehling method was used to prepare cell wall mannan. However, since the mannan molecule was exposed to a strong alkaline solution in the Fehling method, the final form of mannan lacks the *O*‐linked sugar chain. Therefore, the proportion of *O*‐linked sugar chain in the yeast cell wall mannan molecule may be considerably larger than that previously recognized. Therefore, it may be necessary to revalidate the antigenic structure of surface polysaccharides of several *Candida* species other than *C. krusei* by adopting the Benanomicin method established in this report.

In recent studies on the infection mechanism of *Candida* yeasts to the host, mutant strains lacking the *PMT* or *MNT* gene family encoding the protein *O*‐mannosyltransferase or α‐1,2‐mannosyltransferase, respectively, involved in the biosynthesis of *O*‐linked sugar chains are frequently used. In these reports on bioactivities such as adhesion to host cells 30, 31, lethal activity of experimental animals 31, impact on host cellular immune response 32, 33, resistance to antibiotics 34 and formation of biofilm 35, the important roles of *O*‐linked sugar chains in the mannan molecule have been discussed. However, in order to investigate whether the *O*‐linked sugar chain in mannan was directly involved in the *Candida* infection mechanism or the biological activity of mannan molecule, it is necessary to successfully isolate the mannan molecule from the wild‐type pathogen by β‐elimination and utilize the resultant sugar chain as an inhibitor for each biological reaction system. Therefore, to elucidate these mechanisms, the new mannan preparation methods using Benanomicin A established in this report would be of great use.

## Author contributions

HK conceived and supervised the study. HO, AI, YO, and TK performed experiments. TK and HK wrote the manuscript.

## Conflict of interest

The authors declare no conflict of interest.
